# Two single nucleotide polymorphisms in the von Hippel-Lindau tumor suppressor gene in Taiwanese with renal cell carcinoma

**DOI:** 10.1186/1756-0500-7-638

**Published:** 2014-09-12

**Authors:** Wen-Chung Wang, Mei-Hua Tsou, Hui-Ju Chen, Wei-Fang Hsu, Yen-Chein Lai

**Affiliations:** Department of Obstetrics and Gynecology, Jen-Ai Hospital, Taichung, Taiwan Republic of China; Department of Pathology and Laboratory Medicine, Koo Foundation Sun Yat-Sen Cancer Center, Taipei, Taiwan Republic of China; School of Medical Laboratory and Biotechnology, Chung Shan Medical University, No.110, Sec. 1, Chien Kuo N. Road, Taichung, 402 Taiwan Republic of China; Department of Microbiology and Immunology, Chang Gung University, Taoyuan, Taiwan Republic of China

**Keywords:** Loss of heterozygosity, Renal cell carcinoma, Single nucleotide polymorphism, Von Hippel-Lindau tumor suppressor gene

## Abstract

**Background:**

Renal cell carcinoma, a common malignant tumor arising from the kidney, occurs in 3.62 and 1.95 cases per one hundred thousand people among men and women, respectively, in Taiwan each year. Approximately 80% of cases are classified as clear-cell renal cell carcinoma. Inactivation of the von Hippel-Lindau tumor suppressor gene has been implicated in the tumorigenic pathway of renal cell carcinoma. Two single nucleotide polymorphisms, rs779805 and rs1642742, located in the promoter and 3′ untranslated regions of the von Hippel-Lindau gene are informative and implicated in the occurrence of renal cell carcinoma worldwide. The aim of this study is to clarify whether these polymorphisms are associated with renal cell carcinoma in Taiwanese. Genomic DNA was isolated from normal and tumor tissues of 19 renal cell carcinoma patients. The samples were screened for allelic polymorphisms by restriction fragment length polymorphism with *BsaJ* I and *Acc* I digestion. Reconfirmation was carried out by direct sequencing.

**Results:**

Consistent with Knudson’s two-hit theory, AA to AG somatic mutations were observed in rs779805. In addition, loss of heterozygosity in both rs779805 and rs1642742 was demonstrated in 10 out of 15 RCC patients aged 50 or over. The G allele or AG heterozygote frequencies at these two loci were much higher in patient germline DNA when compared with the control group. After adjusting for age, the frequency of the G allele in both loci was much higher for late onset renal cell carcinoma in the Taiwanese population.

**Conclusions:**

Our current results confirmed that the existence of G allele in both rs779805 and rs1642742 in the von Hippel-Lindau tumor suppressor gene is of importance in renal cell carcinoma tumorigenesis. However, more comprehensive and detailed research is needed to address the clinical relevance. Larger sample size is required to determine the exact power of correlation between these two genetic polymorphisms and renal cell carcinoma.

**Electronic supplementary material:**

The online version of this article (doi:10.1186/1756-0500-7-638) contains supplementary material, which is available to authorized users.

## Background

Von Hippel-Lindau (VHL) disease (OMIM 193300) is an autosomal dominantly inherited tumor syndrome affecting 1 in 36,000 in the human population [1]. The most common types of tumors are retinal angioma, cerebellar and spinal hemangioblastoma, clear cell renal cell carcinoma (RCC) and pheochromocytoma [2]. Tumors form when there is biallelic *VHL* tumor suppressor gene inactivation in a two hit model of tumorigenesis [3–5]. The *VHL* tumor suppressor gene is located on the short arm of chromosome 3 mapped to 3p25-3p26 and consists of 639 nucleotides in 3 exons encoding 213 amino acids [6, 7]. Germ-line mutations have been detected in approximately 500 VHL family members including deletions in part of or the whole gene, as well as intragenic point mutations and micro-deletions/insertions [8]. Somatic inactivating mutations have been identified in tumors from VHL-affected [9] and sporadic RCC [6, 10] patients.

The etiology of renal cancer is still not fully understood. Nevertheless, a number of environmental factors have been implicated in sporadic RCC [11]. RCC is the most common malignant renal cancer with around 7.5 cases per 100,000 people, which is equal to about 3% of all adult cancers in Western countries [12]. Most RCCs are classified as clear-cell RCC. More than half of RCC cases are linked to either *VHL* gene mutations or transcriptional repression with hypermethylation in the promoter and first exon regions [13, 14]. Multiple and bilateral RCCs are present in up to 40% of VHL patients. It is highly possible that subjects with VHL disease will develop RCC if they live long enough [15]. As early detection and intervention have reduced the death rate from VHL central nervous complications, bilateral multiple cystic RCC has become the leading cause of death in patients with VHL disease [16]. Recent reports have shown that acquired cystic disease-associated RCC frequently occurs with abnormalities on chromosome 3 in the Taiwanese population [17]. To date, no studies have focused on the association between *VHL* gene malfunction and RCC in Taiwan.

Two single nucleotide polymorphisms (SNPs), rs779805 and rs1642742 involving both A and G, located in the promoter and 3′ untranslated regions of the VHL gene are informative and implicated in the occurrence of RCC worldwide [18–20]. The aim of this study is to clarify whether these polymorphisms are associated with RCC in Taiwanese. We confirmed somatic mutation and loss of heterozygosity (LOH) at these two loci in RCC patients. In addition, a genetic association pilot study using these two SNPs for germline DNA genotyping was conducted to test the potential causality between *VHL* gene dysfunction and RCC susceptibility in the Taiwanese population.

## Methods

### Study subjects

Paraffin-embedded normal and tumor tissue samples from RCC patients were provided by the Tumor Tissue Bank of the Koo Foundation Sun Yat-Sen Cancer Center, which is funded by the National Science and Technology Program for Pharmaceuticals and Biotechnology (#NSC89-2323-B-368-001). The control group consisted of 616 unrelated individuals and has been described in our previous report [21]. DNA sample collection for the *VHL* SNP study protocol was approved by the Institutional Review Board of Chung Shan Medical University Hospital and informed consent was obtained from each subject.

### Preparation of Genomic DNA

The tissues were deparaffinized using standard xylene-deparaffin procedure. Genomic DNA was isolated from normal and tumor tissues using QIAamp DNA kit (Qiagen). The concentration of nucleic acids was estimated by GeneQuant II RNA/DNA calculator (Amersham Pharmacia Biotech). Each genomic DNA sample was adjusted to 100 ng/ml and served as a template for subsequent analyses.

### Polymerase chain reaction (PCR)

*VHL* rs779805A > G and rs1642742A > G were detected on polymerase chain reaction–restriction fragment length polymorphism (PCR-RFLP) assays according to the methods described by Gail et al. and Payne et al. [22, 23]. Slight modifications were made in the PCR primers for rs779805 and rs1642742 as follows: rs779805-F, 5′-AGCCTCGCCTCCGTTACCAC-3′; rs779805-R, 5′-GTAGAGGATGGAACGCGCT-3′; rs1642742-F, 5′-CTGCCCATTAGAGAAGTATTT-3′; rs1642742-R, 5′-AATTCCCACTGAATTACGTATA-3′. PCR fragments were amplified in a Perkin-Elmer 2400 DNA thermal cycler in a final volume of 15 to 30 µl that contained one-fold Qiagen PCR buffer [Tris–HCl, KCl, (NH_4_)_2_SO_4_, 15 mM MgCl_2_; pH 8.7 at 20°C], one-fold Q-solution, 0.015 units/µl *Taq* DNA polymerase supplied from *Taq* DNA polymerase kit (Qiagen), 500 nM for each primer, 200 mM dGTP, dATP, dCTP and dTTP (Promega) and 300 ng/µl template. PCR conditions included initial denaturation for 5 min at 95°C, followed by 35 cycles consisting of 1 min at 95°C, 1 min at 60°C for rs779805 or 50°C for rs1642742, and 2 min at 72°C, with final extension for 10 min at 72°C.

### Digestion of restriction enzyme

An aliquot of 10 µl of PCR product was digested with 2.5 units *BsaJ* I (New England Biolabs) for rs779805 or 2.5 units *Acc* I (New England Biolabs) for rs1642742 in a total volume of 20 µl that contained one-fold NEBuffer 2 or NEBuffer 4. The samples were digested at 60°C for rs779805 or 37°C for rs1642742 for 4 hrs followed by heat inactivation of the restriction enzyme at 80°C for 20 min. After digestion, the products were separated onto 3% agarose gels. To avoid genotyping errors, analysis was repeated at least twice for each sample. Portions of the PCR products were sent for direct sequencing and the data were consistent with the genotyping.

### Direct sequencing

The A/G polymorphisms at nucleotides 19 (rs779805) and 1149 (rs1642742) in the *VHL* gene were analyzed by PCR amplification using published primers and experimental protocols [8, 20, 24]. The PCR products were purified using QIAquick PCR Purification kits (Qiagen). Then, the purified PCR products were sequenced by the cycle-sequencing method with fluorescently labeled dideoxy chain terminators from ABI Prism kit (Applied Biosystems) in an ABI Model 377 automated DNA sequencer. The sequencing primers were the same as those for the preceding PCRs. The nucleotide sequence was confirmed on both strands.

### Statistical analysis

Standard normal deviate (z) test or chi-square test was used to compare the genotype and allelic frequencies between the control and patient groups. *p* values of less than 0.05 were considered statistically significant and *p* values of less than 0.01 were considered highly significant.

## Results

### Somatic changes in rs779805 and rs1642742 in the VHL gene in RCC patients

We compared the DNA from normal and tumor tissues from RCC patients to investigate somatic gene alteration. We observed AA to AG somatic mutations in four RCC cases, aged 50 or over, among a total 12 AA homozygotes (33.3%) at rs779805 (Table 1). All four RCC cases with AA homozygote at rs779805 were aged less than 50 and possessed the same genotype in their tumor tissues. Similar phenomena were not observed for rs1642742.

**Table 1:** **RCC patients with and without somatic mutations of rs779805 and rs1642742 of VHL gene in tumor tissue**

					rs779805		rs1642742	
#	Age	Gender	Stage	Clear cells	Normal	Tumor	Normal	Tumor
With somatic mutations								
1	66	Female	III	Yes	AG	LOH (G)	AG	LOH (G)
2	77	Female	I	Yes	AG	LOH (A)	AG	LOH (A)
3	50	Male	I	Yes	AG	LOH (G)	AG	LOH (G)
4	53	Female	II	Yes	AG	LOH (A)	AG	ND^*^
5	83	Female	I	Yes	AG	ND^*^	AG	LOH (G)
6	51	Male	I	Yes	AG	LOH (G)	AG	LOH (A)
7	70	Female	IV^#^	Yes	AA	AG	AG	LOH (G)
8	51	Male	III	Yes	AA	AG	AA	ND^*^
9	53	Female	I	Yes	AA	AG	AA	ND^*^
10	63	Female	II	Yes	AA	AG	AA	ND^*^
Without somatic mutations								
11	67	Male	I	Yes	AG	ND^*^	AG	ND^*^
12	62	Male	III	Yes	AA	ND^*^	AA	ND^*^
13	45	Male	I	Yes	AA	ND^*^	AA	ND^*^
14	72	Female	II	Yes	AA	ND^*^	AA	ND^*^
15	31	Male	I	Yes	AA	ND^*^	AA	ND^*^
16	33	Male	I	Yes	AA	ND^*^	AA	ND^*^
17	40	Female	I	Yes	AA	ND^*^	AA	ND^*^
18	70	Male	I	Yes	AA	ND^*^	AA	ND^*^
19	76	Male	I	No	AA	ND^*^	AA	ND^*^

^#^metastasis; ^*^ND, no somatic changes were detected.

**Figure 1:**
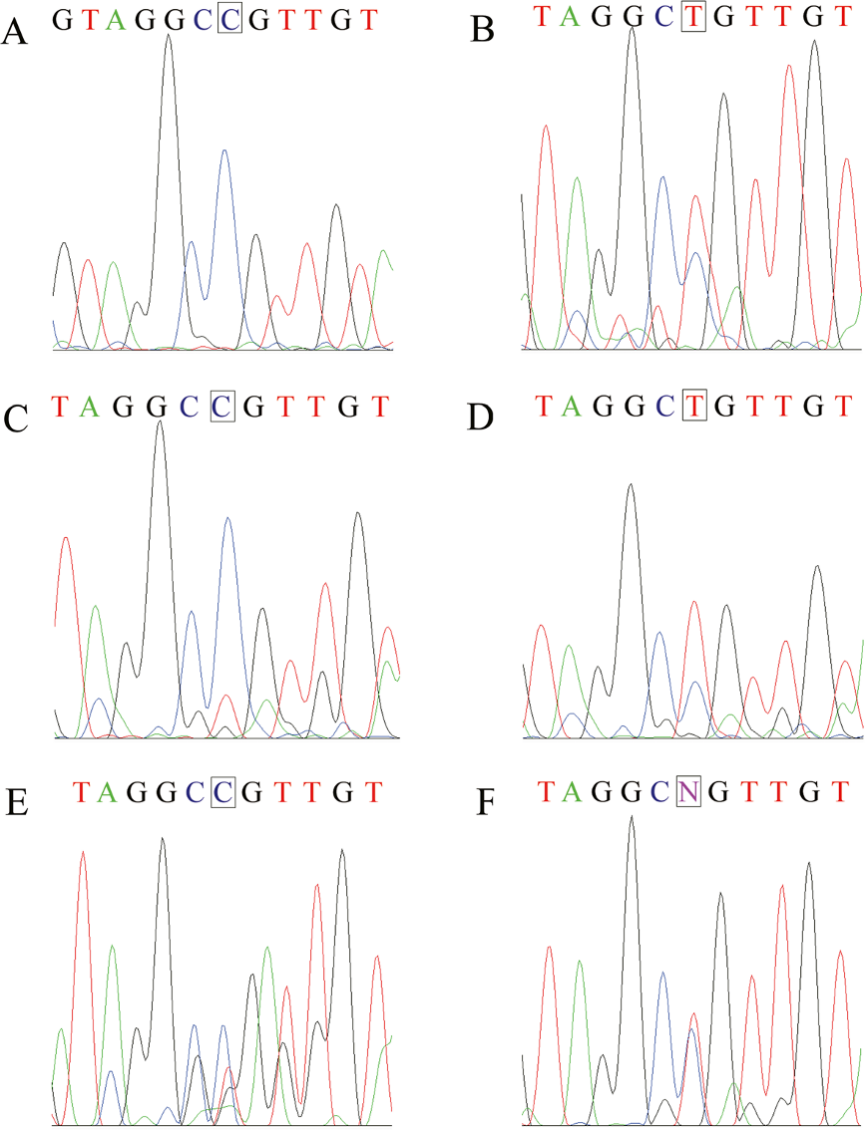
**Partial sequencing chromatograms of the VHL gene containing rs779805 in reverse direction from tumor tissues.** The rs779805 is represented by a rectangular frame. **(A)**, **(C)**, and **(E)** represent examples of loss of heterozygosity (LOH) of the A allele at rs779805 of *VHL* gene in RCC patients (#1, #3, and #6 in Table 1). **(B)** and **(D)** represent loss of the G allele (#2 and #4). **(F)** is a heterozygote with two almost equal bands (#11).

**Figure 2:**
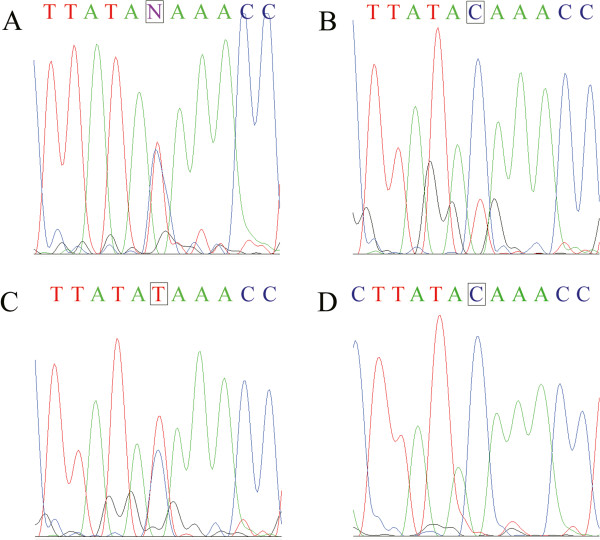
**Partial sequencing chromatograms of the VHL gene containing rs1642742 in reverse direction.** The rs1642742 is represented by a rectangular frame. Normal tissue **(A)** and tumor tissue **(B)** from heterozygous individual #1 in Table 1 show LOH. **(B)** and **(D)** represent examples of LOH of the A allele at rs1642742 of *VHL* gene in the tumor tissues from RCC patients (#1 and #7). **(C)** represents loss of the G allele (#2).

In addition to AA to AG somatic mutations, LOH was identified in RCC patients with AG heterozygote at rs779805 and rs1642742. PCR-RFLP analysis of heterozygotes of A allele and G allele normally results in two equal bands. However, there were differences between normal and tumor tissues (Additional file 1: Figure S1). We compared the relative extent of these two bands from tumor tissue with that from normal tissue and obtained a ratio (R). LOH was considered positive when R was larger than 1.25 or smaller than 0.8. There were seven AG heterozygotes at rs779805 (out of 19 RCC cases). Five of them (71.4%) showed LOH (Table 1). In addition to digestion of restriction enzyme, we further confirmed LOH by DNA sequencing and the sequencing patterns were significantly altered at rs779805 (Figure 1), with three demonstrating G alleles (Figure 1A, C, E). In addition, there were eight AG heterozygotes at rs1642742 with six (75.0%) showing LOH (Table 1). Four of them possessed G alleles (Figure 2C, D) and the remaining two possessed A alleles (Figure 2C). There were seven AG heterozygotes at rs779805. These seven individuals also demonstrated AG heterozygote at rs1642742. Four out of these seven (57.1%) showed LOH in both loci. All RCC patients with LOH were aged 50 or over.

Consistent with Knudson’s two-hit theory, AA to AG somatic mutations were present in rs779805 and LOH was present in both rs779805 and rs1642742 in 10 out of 15 RCC patients aged 50 or over (Table 1). To test whether these polymorphisms are associated with predisposition for RCC in Taiwanese, a genetic association pilot study was conducted using these two SNPs for germline DNA genotyping.

### AG heterozygote frequencies in both rs779805 and rs1642742 increase in the germline DNA of RCC patients

We have previously reported that the G allelic frequencies in both rs779805 and rs1642742 of *VHL* gene in healthy subjects from Taiwan are much lower than in the European population [21]. Here, we examined the genotype distributions and G allelic frequencies at these two SNPs in normal tissues, which represented germline DNA, of RCC patients from Taiwan. In normal control subjects (n = 616), the frequencies of the G allele at rs779805 and rs1642742 were 0.130 and 0.133, respectively, whereas in RCC patients (n = 19) the frequencies of the G allele at rs779805 and rs1642742 were 0.184 and 0.211, respectively (Table 2). The G allelic frequencies increased 41.5% (0.184/0.130) and 58.6% (0.211/0.133), respectively, in RCC patients. In normal control subjects, the observed heterozygosities for rs779805 and rs1642742 were 0.214 and 0.218, respectively, whereas in RCC patients the observed heterozygosities for rs779805 and rs1642742 were 0.368 and 0.421, respectively (Table 2). The frequencies of heterozygous genotype increased 71.9% (0.368/0.214) and 93.1% (0.421/0.218), respectively, in RCC patients. Similar phenomena were observed when we compared frequencies of the G allele carriage (AG + GG), with increases from control level of 0.237 to 0.368 for rs779805 (55.3% increase) and from 0.242 to 0.421 for rs1642742 (74.0% increase).

**Table 2:** **Comparison of the frequency distributions of rs779805 and rs1642742 of VHL gene in the germline DNA of RCC patients with normal controls**

	rs779805		rs1642742	
	19 RCCs ^#^	616 controls ^#^	19 RCCs ^#^	616 controls ^#^
*Genotype distribution*				
AA	12 (63.16%)	470 (76.30%)	11 (57.89%)	467 (75.81%)
AG	7 (36.84%)	132 (21.43%)	**8 (42.10%)**	**134 (21.75%)**
GG	0	14 (2.27%)	0	15 (2.44%)
*Allele frequency*				
A allele	31 (81.58%)	1072 (87.01%)	30 (78.95%)	1068 (86.69%)
G allele	7 (18.42%)	160 (12.99%)	8 (21.05%)	164 (13.31%)
*Homozygous genotype*				
(AA or GG genotype)	12 (63.16%)	484 (78.57%)	11 (57.89%)	482 (78.25%)
*Heterozygous genotype*				
(AG genotype)	7 (36.84%)	132 (21.43%)	**8 (42.01%)**	**134 (21.75%)**
*Allele carriage frequency*				
A allele (AA+AG)	19 (100%)	602 (97.73%)	19 (100%)	601 (97.56%)
G allele (AG+GG)	7 (36.84%)	146 (23.70%)	8 (42.10%)	149 (24.19%)

^#^Data presented as number of cases and percentage; bold text means statistical significance was reached, p < 0.05, in the comparison of germline DNA between RCC patients and controls.

A positive correlation between RCC and G variant has been indicated [21], suggesting that the G variant is a lethal genetic mutation resulting in the lack of GG homozygote in the RCC patients in the current study. That is to say, if the G allele or AG heterozygote frequency increases, chance of somatic changes and susceptibility to RCC increase. However, further studies with a larger sample size are needed to verify this hypothesis.

### Patient characteristics in relation to rs779805 and rs1642742 genotypes in patients with RCC

The main characteristics of the patient population are shown in Table 3. Age (≥50, < 50), sex, clear cell type (yes/no), and tumor stage (early, stage I; late, stage II to IV) were dichotomous variables based on Moore's work [25]. Delayed onset is a key feature of sporadic cancers. On multivariate analysis, age was an important factor in the genotype distributions of both rs779805 and rs1642742 (Table 3). For all RCC patients, the observed frequencies of heterozygosity for rs779805 and rs1642742 were 0.368 and 0.421, respectively. In all four RCC cases aged less than 50, AA homozygote was observed at both rs779805 and rs1642742. Among the 15 RCC cases aged 50 or over, the observed frequencies of heterozygosity for rs779805 and rs1642742 were 0.467 and 0.533, respectively. The frequencies of heterozygous genotype increased 26.9% (0.467/0.368) and 26.6% (0.533/0.421), respectively, in RCC patients aged 50 or over. These data suggested that the frequencies of AG heterozygote at both rs779805 and rs1642742 in the *VHL* tumor suppressor gene are much higher for late onset RCC in the Taiwanese population. Somatic changes in rs779805 and rs1642742 were only observed in RCC patients aged 50 or over (Table 1).

**Table 3:** **Analysis of patient characteristics in relation to rs779805 and rs1642742 genotypes in patients with RCC**

		rs779805		rs1642742	
	All patients ^#^	AA genotype ^#^	AG genotype ^#^	AA genotype ^#^	AG genotype ^#^
Total	19	12 (63.16%)	7 (36.84%)	11 (57.89%)	8 (42.10%)
Age					
< 50 years	4	4 (100%)	0 (0%)	4 (100%)	0 (0%)
≥50 years	15	**8 (53.33%)**	**7 (46.67%)**	**7 (46.67%)**	**8 (53.33%)**
Gender					
Male	10	7 (70.00%)	3 (30.00%)	7 (70.00%)	3 (30.00%)
Female	9	5 (50.00%)	4 (40.00%)	**4 (40.00%)**	**5 (50.00%)**
Tumor stage					
I	12	7 (58.33%)	5 (41.67%)	7 (58.33%)	5 (41.67%)
II+III+IV	7	5 (71.43%)	2 (28.57%)	4 (57.14%)	3 (42.86%)
Clear cells					
No	1	1 (100%)	0 (0%)	1 (100%)	0 (0%)
Yes	18	11 (61.11%)	7 (38.89%)	**10 (55.56%)**	**8 (44.44%)**

^#^Data presented as number of cases and percentage; bold text means statistical significance was reached, p < 0.05, in the comparison of genotype distributions between two sub-groups.

Adjustment for tumor stage (I vs II + III + IV) did not significantly affect the genotype distributions (Table 3). Gender and clear cell type were factors in the genotype distributions of both rs779805 and rs1642742 (Table 3). Among female RCC cases, the frequencies of heterozygous genotype increased from 0.368 for total patients to 0.400 for rs779805 (8.7% increase) and from 0.421 for total patients to 0.500 for rs1642742 (18.8% increase). Among clear cell RCC cases, the frequencies of heterozygous genotype increased from 0.368 for total patients to 0.389 for rs779805 (5.7% increase) and from 0.421 for total patients to 0.444 for rs1642742 (5.4% increase).

## Discussion

All increases in rs1642742 shown in Table 2 and Table 3 are higher than in rs779805, indicating that rs1642742 is a more sensitive risk factor for sporadic RCC. However, further studies with a larger sample size are needed to clarify this phenomenon. Statistical significance was reached with just 19 samples for AG heterozygote frequencies in rs1642742 in the comparison of germline DNA between RCC patients and controls using chi-square test (χ^2^ = 4.397, *p* = 0.036; bold in Table 2). Statistical significance was also reached in both loci between older and total RCC cases (χ^2^ = 5.132, *p* = 0.023* for rs779805; χ^2^ = 7.948, *p* = 0.0048* for rs1642742; bold in Table 3). AG heterozygote at rs1642742 was significantly associated with female gender and clear cell type (*p* = 0.018* and *p* = 0.028*; bold in Table 3). However, the small sample size of 19 patients in this study may not be enough to make broad generalizations.

Among the RCC patients in this study, the G allele carriage in rs779805 showed a slightly increasing trend. The location of rs779805 is 128 bp upstream from the major transcription-starting site within the promoter region of the *VHL* gene and its 5’ adjacent nucleotide is C. The somatic mutations from AA homozygote to AG heterozygote at rs779805 create a new CpG island that can be methylated to suppress gene expression. A similar rationale can be applied to RCC patients with germline AG heterozygote at rs779805. These somatic mutations were observed in four out of 15 RCC patients aged 50 or over (Table 1). The accumulation of somatic mutations becomes more evident late in life. Cancer may develop after shutting down of the function of *VHL* gene, when A allele is changed to G allele, followed by methylation of the CpG island.

Among the haploinsufficient tumor suppressor genes, it is possible that one allele is not able to function sufficiently [26]. Previous evidence regarding the Dmp1 gene has shown the effect of haploinsufficiency on tumorigenesis [27]. The differences in the distributions of AA homozygote and AG heterozygote at rs779805 and rs1642742 in this study may be a case of haploinsufficiency. GG homozygote was not observed in our RCC samples. A possible explanation is that GG homozygote causes early cell death before clinical cancer can develop.

Our results were consistent with those of previous reports, namely that *VHL* gene dysfunction is observed in sporadic RCC, as well as in hereditary cases [13, 28, 29]. Three polymorphic markers at 3p25-p26 have been examined for LOH in RCC [18]. Patients with sporadic RCC have been shown to have LOH in two SNPs in *VHL* gene [19, 20] and present with a biallelic polymorphism with either an A or a G at positions 19 (rs779805) and 1149 (rs1642742) in the nucleotide sequence [22, 23]. From a series of studies performed in Western countries, the G allele carriage at rs779805 and rs1642742 is an important risk factor for clear cell RCC [25], consistent with our findings. In this study, it was found that LOH at rs779805 and rs1642742 in the *VHL* gene in RCC patients manifests only late in life (Table 1). Our results were also consistent with previous findings of LOH in most RCC patients with AG heterozygosity [19, 20]. The current findings agree with Knudson’s two-hit theory that, in addition to gene mutation and hypermethylation, LOH is one of the major mechanisms resulting in tumor suppressor gene (such as *VHL* gene) inactivation [3–5]. LOH in tumor suppressor gene has been observed in most populations. However, ethnic differences may affect the frequency of occurrence [30]. LOH was considered positive when R was larger than 1.25 or smaller than 0.8 because even partial inactivation of tumor suppressors can contribute to tumorigenesis [5].

Linehan et al. reported that *VHL* gene inactivation might occur in the early stage of tumorigenesis in RCC [31]. In this study, most RCC patients with AG heterozygosity were identified as having LOH. LOH in *VHL* gene, therefore, is a beneficial tool for early diagnosis of RCC, as well as for monitoring of recurrence in RCC patients [31]. *VHL* gene polymorphism/association information obtained from this study is useful for further genetic RCC studies and for facilitating the development of new treatments. The results of this study can also be used to establish genetic diversity panels for the *VHL* tumor suppressor gene in the local population. Although further studies with larger sample sizes are needed to address the details of the association of *VHL* SNPs with RCC, as well as ethnic variation, these SNPs may be useful genetic tumor markers for the molecular diagnostics of clear cell RCC in the elderly population in Taiwan.

## Conclusions

In the current study, we examined the association of two allelic VHL gene polymorphisms, rs779805 and rs1642742, with RCC in patients from Taiwan. By comparing the DNA from normal and tumor tissues, AA to AG somatic mutations at rs779805 and loss of heterozygosity at both rs779805 and rs1642742 in 10 out of 15 RCC patients aged 50 or over were observed. In comparison with the healthy control group, the G allele and AG heterozygote frequencies at these two loci were much higher in the patient germline DNA than in the control group on genetic association pilot study, especially for rs1642742 and late onset RCC, suggesting that the existence of G allele at both rs779805 and rs1642742 is of importance in RCC tumorigenesis. However, more comprehensive and detailed research is needed to address the clinical relevance. Larger sample size is required to determine the exact power of correlation between these two genetic polymorphisms and RCC.

## Electronic supplementary material

Additional file 1: Figure S1: RFLP analysis was used to screen for the allele genotype of rs779805 by BsaJ I digestion methods. Examples of rs779805 identified in three RCC patients. Lanes 1, 3, and 5 are normal tissues; Lanes 2, 4, and 6 are tumor tissues. The upper band represents A allele in uncleaved 101-bp PCR fragments. The lower band represents G allele with cleavage of 101-bp PCR fragments into fragments of 83-bp and 18-bp in length. The 18-bp fragments were run off the gel. (DOC 153 KB)

## Abbreviations

VHL: Von Hippel-Lindau
RCC: Renal cell carcinoma
RFLP: Restriction fragment length polymorphism
SNP: Single nucleotide polymorphism
LOH: Loss of heterozygosity
PCR: Polymerase chain reaction.
