# Supplementary material for: Two single nucleotide polymorphisms in the von Hippel-Lindau tumor suppressor gene in Taiwanese with renal cell carcinoma
Source: BMC Res Notes. 2014 Sep 12;7:638. doi: 10.1186/1756-0500-7-638 (PMC4168206; doi:10.1186/1756-0500-7-638)
Supplement: Supplementary file 1 — Additional file 1: Figure S1: RFLP analysis was used to screen for the allele genotype of rs779805 by BsaJ I digestion methods. Examples of rs779805 identified in three RCC patients. Lanes 1, 3, and 5 are normal tissues; Lanes 2, 4, and 6 are tumor tissues. The upper band represents A allele in uncleaved 101-bp PCR fragments. The lower band represents G allele with cleavage of 101-bp PCR fragments into fragments of 83-bp and 18-bp in length. The 18-bp fragments were run off the gel. (DOC 153 KB) [file 13104_2013_3159_MOESM1_ESM.doc]

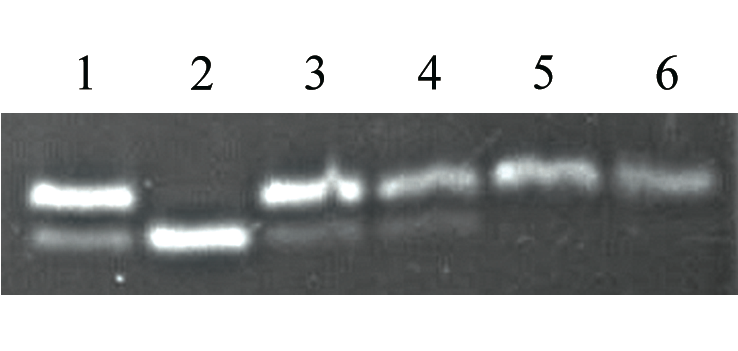


Supplemental Figure 1. RFLP analysis was used to screen for the allele genotype of rs779805 by BsaJ I digestion methods. Examples of rs779805 identified in three RCC patients. Lanes 1, 3, and 5 are normal tissues; Lanes 2, 4, and 6 are tumor tissues. The upper band represents A allele in uncleaved 101-bp PCR fragments. The lower band represents G allele with cleavage of 101-bp PCR fragments into fragments of 83-bp and 18-bp in length. The 18-bp fragments were run off the gel.
